# Adolescent pregnancy at antiretroviral therapy (ART) initiation: a critical barrier to retention on ART

**DOI:** 10.1002/jia2.25178

**Published:** 2018-09-18

**Authors:** Harriet Nuwagaba‐Biribonwoha, Agnes N Kiragga, Constantin T Yiannoutsos, Beverly S Musick, Kara K Wools‐Kaloustian, Samuel Ayaya, Hilary Wolf, Emmanuel Lugina, John Ssali, Elaine J Abrams, Batya Elul, the International epidemiology Databases to Evaluate AIDS (IeDEA) East Africa Collaboration

**Affiliations:** ^1^ Mailman School of Public Health ICAP at Columbia University New York NY; ^2^ Department of Epidemiology Columbia University Mailman School of Public Health New York NY; ^3^ Research Department Infectious Diseases Institute College of Health Sciences Makerere University Kampala Uganda; ^4^ Fairbanks School of Public Health Indiana University Indianapolis IN; ^5^ School of Medicine Indiana University Indianapolis IN; ^6^ Academic Model Providing Access to Healthcare (AMPATH) Moi University Eldoret Kenya; ^7^ Department of Pediatrics University of Maryland School of Medicine Baltimore MD; ^8^ Ocean Road Cancer Institute Dar es Salaam Tanzania; ^9^ Masaka Regional Referral Hospital Masaka Uganda; ^10^ Vagelos College of Physicians and Surgeons Columbia University New York NY

**Keywords:** antiretroviral therapy (ART), pregnancy, adolescents, Africa, ART retention, HIV outcomes

## Abstract

**Introduction:**

Adolescence and pregnancy are potential risk factors for loss to follow‐up (LTFU) while on antiretroviral therapy (ART). We compared adolescent and adult LTFU after ART initiation to quantify the impact of age, pregnancy, and site‐level factors on LTFU.

**Methods:**

We used routine clinical data for patients initiating ART as young adolescents (YA; 10 to 14 years), older adolescents (OA; 15 to 19 years) and adults (≥20 years) from 2000 to 2014 at 52 health facilities affiliated with the International epidemiology Databases to Evaluate AIDS (IeDEA) East Africa collaboration. We estimated cumulative incidence (95% confidence interval, CI) of LTFU (no clinic visit for ≥6 months after ART initiation) and identified patient and site‐level correlates of LTFU, using multivariable Cox proportional hazards models for all patients as well as individual age groups.

**Results:**

A total of 138,387 patients initiated ART, including 2496 YA, 2955 OA and 132,936 adults. Of these, 55%, 78% and 66%, respectively, were female and 0.7% of YA, 22.3% of OA and 8.3% of adults were pregnant at ART initiation. Cumulative incidence of LTFU at five years was 26.6% (24.6 to 28.6) among YA, 44.1% (41.8 to 46.3) among OA and 29.3% (29.1 to 29.6) among adults. Overall, compared to adults, the adjusted hazard ratio, aHR, (95% CI) of LTFU for OA was 1.54 (1.41 to 1.68) and 0.77 (0.69 to 0.86) for YA. Compared to males, pregnant females had higher hazard of LTFU, aHR 1.20 (1.14 to 1.27), and nonpregnant women had lower hazard aHR 0.90 (0.88 to 0.93). LTFU hazard among the OA was primarily driven by both pregnant and nonpregnant females, aHR 2.42 (1.98 to 2.95) and 1.51 (1.27 to 1.80), respectively, compared to men. The LTFU hazard ratio varied by IeDEA program. Site‐level factors associated with overall lower LTFU hazard included receiving care in tertiary versus primary‐care clinics aHR 0.61 (0.56 to 0.67), integrated adult and adolescent services and food ration provision aHR 0.93 (0.89 to 0.97) versus nonintegrated clinics with food ration provision, having patient support groups aHR 0.77 (0.66 to 0.90) and group adherence counselling aHR 0.61 (0.57 to 0.67).

**Conclusions:**

Older adolescents experienced higher risk of LTFU compared to YA and adults. Interventions to prevent LTFU among older adolescents are critically needed, particularly for female and/or pregnant adolescents.

## 1 Introduction

Nearly two million adolescents (10 to 19 years) are living with HIV in sub‐Saharan Africa, the most significantly HIV impacted region of the world 1. Due to the anticipated increase in absolute population numbers of adolescents and youth 15 to 24 years, called the youth bulge 2, the number of new HIV infections among adolescents is expected to increase by 2030 1, making this a critically important group to focus on in the HIV response. The use of antiretroviral (ART) therapy is associated with less morbidity and mortality among persons living with HIV (PLHIV), and declining mortality trends have been observed with ART scale‐up globally 3. However, loss to follow‐up (LTFU) after ART initiation can potentially reverse these gains.

Some studies have compared rates of LTFU after ART initiation between adolescents and youth, and adults with mixed results: some have reported higher LTFU among the adolescents compared to adults 4, while others showed similar or better outcomes 5, 6, 7. However, these studies were limited by inadequate disaggregation of the adolescent age group. Adolescents (10 to 19 years) represent a mix of both a younger (10 to 14 years) and older (15 to 19 years) population, who are at different developmental stages and may have vastly different family support and patterns of accessing health care. This may in turn drive different ART retention behaviours. A better understanding of the ART retention patterns of younger and older adolescents is needed in order to appropriately target retention interventions.

Additionally, pregnancy at ART initiation is increasingly being associated with high LTFU after ART initiation in multiple cohorts, particularly as countries adopt lifelong ART for pregnant and breastfeeding women (Option B+) 8, 9, 10, 11, 12. Reasons for this may include individual factors related to child care, relationships with partners and family, migration postdelivery, stigma, and social barriers 13. Additionally, structural barriers such as limited access to services, limited integration of ART in postnatal services and other health system challenges may impact ART retention 13. These barriers are likely to be amplified among pregnant adolescents. To date, there is limited exploration of the impact of pregnancy on LTFU among adolescents who initiate ART.

In order to explore and quantify the impact and interactions between age, pregnancy, and site‐level factors on LTFU among adolescents initiating ART in East Africa, we undertook a multicountry analysis which compared the rates and factors associated with LTFU among young adolescents (YA, 10 to 14 years), older adolescents (OA, 15 to 19 years) and adults (20 years and older).

## 2 Methods

### 2.1 Study design

We conducted a retrospective cohort analysis of routinely collected clinical data from patients initiating ART at health facilities affiliated with the East Africa region of the International epidemiology Databases to Evaluate AIDS (IeDEA) collaboration 14. This study was approved by the Indiana and Columbia University Institutional Review Boards (IRBs) and all regulatory bodies affiliated with the sites contributing data to this analysis. Only de‐identified data were transferred to the East African IeDEA Regional Data Center, and as such, all regulatory bodies waived the written consent requirement.

### 2.2 Study population

Individual patient‐level data were included in this analysis if the patient was treatment naïve at enrolment, initiated ART between February 2000 and December 2014 and was at least 10 years of age at ART initiation. Women initiating ART solely for prevention of mother‐to‐child transmission of HIV (PMTCT) rather than for clinical indications were excluded from this analysis. Based on the age of ART initiation, age groups were defined as young adolescents (10 to 14 years), older adolescents (15 to 19 years) or adults (≥20 years).

### 2.3 Setting and standard of care

A total of 52 health facilities representing six programmes in Kenya, Tanzania, and Uganda were included in this analysis. These sites represented a mixture of private and public facilities; primary, secondary and tertiary levels of care; and urban and rural healthcare facilities. In most sites, adolescents and adults were seen in the same clinic. However, models of service delivery varied by site 15. Clinical care was provided in accordance with local or national guidelines which were generally consistent with the WHO guidelines of the period. At enrolment, adolescents and adults were assessed for ART eligibility based on clinical stage (WHO/CDC) and CD4 criteria. Prior to 2011, the criteria for ART initiation were based on variations of the 2006 WHO treatment guidelines 16; all patients with CD4 count <200 cells/μL or WHO Stage 4, pregnant women at CD4 count <350 cells/μL and WHO Stage 3; with one programme treating all individuals with WHO stage 3 or 4 disease. From 2011 onwards, health facilities gradually adopted the 2010 WHO treatment guidelines 17 recommending that all patients (including pregnant women) with a CD4 count <350 cells/μL or WHO Stage 3 or 4 be initiated on ART. The exception was the Academic Model Providing Access to Healthcare (AMPATH) western Kenya programme, which utilized a CD4 count threshold of 500 cells/μL for 10 to 14 year olds during this period.

### 2.4 Data collection and management

As part of routine clinical care, patient characteristics (age, sex) and clinical status (WHO stage, CD4 count), ART regimen and outcomes (e.g. transfer out, LTFU and death) were captured on paper‐based forms at enrolment and follow‐up visits. Data clerks within the programme entered these data into a site‐specific electronic medical record system and conducted quality and logic checks following standardized local data cleaning protocols. Data were deidentified and merged across sites by IeDEA East Africa regional data managers who performed additional data cleaning and validation. The date of database closure varied with three sites leaving the IeDEA consortium during the latter half of 2010, one site contributing data through March 2012, and the remaining sites contributing data through mid to late 2014. A structured site assessment was completed at each site in 2009 and 2014, and gathered information on location and type of clinic, as well as the type of services offered 15.

### 2.5 Data analysis

The outcome of interest was LTFU following ART initiation. Patients were considered LTFU if they did not have a clinic visit for ≥6 months prior to database closure and were not documented as having died (mortality) or transferred out. Patients who had no return visit after ART initiation were randomly imputed days of follow‐up between one and fourteen days, to allow for inclusion of their data in the survival analysis. The imputation model followed a simple uniform distribution for the days of follow‐up. The patient‐level variables of interest were age, CD4 count and WHO stage, calendar year and pregnancy status, all at ART initiation. Using age at ART initiation, patients were categorized as young adolescents 10 to 14 years (YA), older adolescents 15 to 19 years (OA) or adults ≥20 years. CD4 count at ART initiation was defined as the most proximal CD4 count obtained within three months prior to ART initiation and up to seven days after. WHO stage at ART start was defined as the maximum WHO stage documented prior to ART initiation or the first documented within 60 days after the start of treatment. In a number of cases, CDC class was used to reflect disease severity. This was mapped to WHO stage in these analyses as follows: CDC class N→WHO stage 1, A→2, B→3 and C→4. Pregnancy at ART initiation was defined as a recorded pregnancy within two months before or after ART initiation date; or a documented estimated or actual date of delivery (according to available data) which resulted in an actual or estimated date of conception which in turn identified a pregnancy prior to ART‐initiation.

We compared demographic characteristics by age group using descriptive statistics. We estimated the cumulative incidence of LTFU, using the Aalen & Johansen estimator which accounts for competing risks 18, considering mortality as a competing risk. Estimates were provided at six months and annually for five years, in line with routine progress review time points. The effect of patient‐level and site‐level factors on LTFU after ART initiation was assessed using cause‐specific hazards models, with censoring at the time of death or the end of follow‐up. Site‐level variables such as level of care, location of health facility, support services offered and type of programme were included in order to control for unmeasured factors within each model of health service delivery, and generate adjusted hazard ratios (aHR) for LTFU after ART initiation. Due to variable effect on patient retention of adolescent and adult service integration and provision of food rations, a variable was created to account for these two factors together. Models were run for all patients and then individually by age group. All patient‐ and site‐level variables described above were introduced in multivariable models and variables significant at the 5% level were retained. All analyses were performed using STATA 14.2 (Statacorp, College Station, TX, USA).

## 3 Results

Between 2000 and 2014, 211,362 patients age 10 and older were enrolled into East Africa programs, of whom 138,387 (65. 5%) initiated ART. Of these, 2496 (1.8%) were YA, 2955 (2.1%) were OA and 132,936 (96.1%) were adults. The majority of patients starting ART were female, including 54.7% of YA, 77.8% OA and 66.2% adults (Table 1). The median age (inter‐quartile range (IQR)) at ART initiation was 12.5 years (IQR 11.4 to 13.7) among YA, 18.3 years (IQR 16.9 to 19.3) among OA and 35.8 years (IQR 29.8 to 43.3) among adults. CD4 counts at ART initiation were available for 66.8% of YA, 64.1% of OA and 68.4% of adults, with a median CD4 count of 186 cells/μL (IQR 56–343), 216 cells/μL (IQR 85 to 387) and 154 cells/μL (IQR 67 to 252) for YA, OA and adults, respectively. Of the total population initiating ART, pregnancy at ART initiation was documented in 0.7% of YA, 22.3% OA and 8.3% adults. The majority of patients were enrolled in the AMPATH program. Distribution of the study population by site characteristics among YA, OA and adults was similar (Table 2). Half the health facilities in this analysis were primary health facilities (50.0%), and nearly half (42.3%) offered combined adolescent and adult services.

**Table 1:** Demographic and clinical characteristics of adolescents and adults initiating ART in IeDEA East Africa

Variable	Young adolescents (10 to 14 years) N = 2496	Older adolescents (15 to 19 years) N = 2955	Adults (20+ years) N = 132,936
Country, n (%)			
Kenya	2069 (82.9)	2376 (80.4)	106,292 (80.0)
Tanzania	174 (7.0)	137 (4.6)	9093 (6.8)
Uganda	253 (10.1)	442 (15.0)	17,551 (13.2)
Female sex, n (%)	1364 (54.7)	2300 (77.8)	87,950 (66.2)
Pregnant at ART initiation	17 (1.2)	658 (28.3)	11,054 (12.6)
Gender and pregnacy status			
Males	1132 (45.3)	655 (22.2)	44,986 (33.8)
Nonpregnant females	1347 (54.0)	1642 (55.6)	76,896 (57.8)
Pregnant females	17 (0.7)	658 (22.3)	11,054 (8.3)
Age: ART start, years			
Median (IQR)	12.5 (11.4–13.7)	18.3 (16.9–19.3)	35.8 (29.8–43.3)
CD4 cell count at ART start, cells/μL; median (IQR)	186 (56–343)	216 (85–387)	154 (67–252)
CD4 cell count at ART start, cells/μL			
<100	559 (22.4)	529 (17.9)	31,461 (23.7)
100 to 199	312 (12.5)	361 (12.2)	26,367 (19.8)
200 to 349	390 (15.6)	461 (15.6)	22,988 (17.3)
≥350	406 (16.3)	542 (18.3)	10,135 (7.6)
Missing	829 (33.2)	1062 (35.9)	41,985 (31.6)
WHO stage at ART start			
III/IV	476 (19.1)	842 (28.5)	57,688 (43.4)
I/II	549 (22.0)	1678 (56.8)	63,947 (48.1)
Missing	1471 (58.9)	435 (14.7)	11,301 (8.5)
WHO/CDC stage at ART start			
III/IV/B/C	1141 (45.7)	911 (30.8)	57,691 (43.4)
I/II/N/S	944 (37.8)	1697 (57.4)	63,944 (48.1)
Missing	411 (16.5)	347 (11.7)	11,301 (8.5)
Year of ART initiation			
2000 to 2004	109 (4.4)	77 (2.6)	4819 (3.6)
2005 to 2009	1081 (43.3)	914 (30.9)	59,004 (44.4)
2010 to 2012	888 (35.6)	1087 (36.8)	45,434 (34.2)
2013 to 2014	418 (16.8)	877 (29.7)	23,679 (17.8)

IeDEA, International epidemiology Databases to Evaluate AIDS; IQR, inter‐quartile range.

**Table 2:** Distribution of the adolescent and adult population initiating ART by site‐level variables in IeDEA East Africa

	Number of health facilities N = 52	Young adolescents N = 2496	Older adolescents N = 2955	Adults N = 132,936
	n (%)	n (%)	n (%)	n (%)
Participating institutions				
AMPATH	36 (69.2)	1748 (70.0)	1652 (55.9)	91,848 (69.1)
FACES	9 (17.3)	320 (12.8)	717 (24.3)	14,147 (10.6)
Masaka	1 (1.9)	203 (8.2)	390 (13.2)	13,694 (10.3)
MTCT‐PLUS	3 (5.8)	18 (0.7)	11 (0.4)	1131 (0.9)
NACP	2 (3.8)	174 (7.0)	137 (4.6)	9093 (6.8)
Rakai	1 (1.9)	33 (1.3)	48 (1.6)	3023 (2.3)
Level of care				
Primary	26 (50.0)	708 (28.4)	828 (28.0)	33,528 (25.2)
Secondary	21 (40.4)	1066 (42.7)	1349 (45.7)	60,586 (45.6)
Tertiary	5 (9.6)	722 (28.9)	778 (26.3)	38,822 (29.2)
Type of clinic				
Private	4 (7.7)	134 (5.4)	280 (9.5)	4188 (3.2)
Public	48 (92.3)	2362 (94.6)	2675 (90.5)	128,748 (96.9)
Clinic setting^a^				
Integrated: adults and adolescents in combined or family clinics	22 (42.3)	1090 (43.7)	1638 (55.4)	61,108 (46.0)
Separate clinics: adults and adolescents in separate clinics	11 (21.2)	1247 (50.0)	1131 (38.3)	64,704 (48.7)
Nutritional support^a^				
Food rations provided	23 (44.2)	1858 (74.4)	2007 (67.9)	95,858 (69.3)
No food rations	10 (19.2)	479 (19.2)	762 (25.8)	29,954 (22.5)
Clinics setting and nutritional support^a^				
Integrated clinics with food rations	12 (23.1)	611 (24.5)	876 (29.6)	29,954 (22.5)
Integrated clinics without food rations	10 (19.2)	479 (19.2)	762 (25.8)	31,154 (23.4)
Separate clinics with food rations	11 (21.2)	1247 (50.0)	1131 (38.3)	64,704 (48.7)
Location				
Rural	17 (32.7)	303 (12.1)	383 (13.0)	17,003 (12.8)
Urban	20 (38.5)	1181 (47.3)	1297 (43.9)	57,067 (42.9)
In between	15 (28.9)	1012 (40.5)	1275 (43.2)	58,866 (44.3)
Support services provided^a^				
Support groups	31 (59.6)	2287 (91.6)	2707 (97.6)	121,906 (91.7)
Group adherence counselling	29 (55.8)	1842 (73.8)	2361 (79.9)	100,059 (75.3)
Peer educator programme	24 (46.2)	1832 (73.4)	2255 (76.3)	96,582 (72.7)
Outreach services for missed visits	30 (57.7)	2027 (81.2)	2278 (77.1)	105,001 (79.0)

IeDEA, International epidemiology Databases to Evaluate AIDS.

a 19 (36.5%) clinics were missing data on clinic setting, nutritional support and support services, and these included 159 (6.4%) YA, 186 (6.3%) OA and 7124 (5.4%) adults.

### 3.1 Cumulative incidence of LTFU after ART initiation

**Figure 1:**
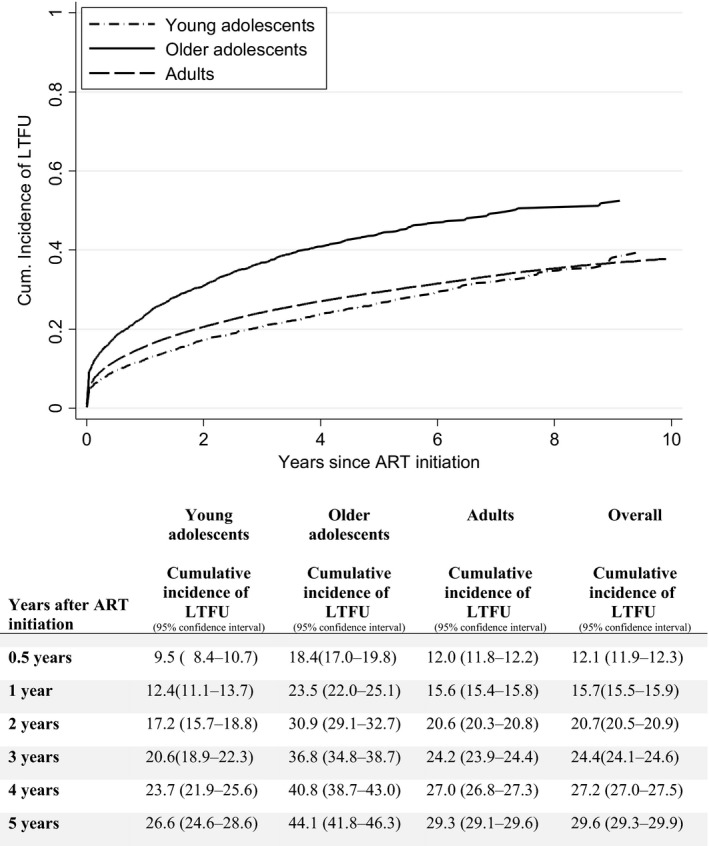
Cumulative incidence of LTFU after ART initiation among YA, OA and adults in IeDEA East Africa, with death as a competing event. LTFU, loss to follow‐up; IeDEA, International epidemiology Databases to Evaluate AIDS; ART, antiretroviral therapy.

The cumulative incidence of LTFU was highest among OA (Figure 1). At six months after ART initiation, 18.4% (95% confidence interval (CI) 17.0 to 19.8) of OA were lost to follow‐up, compared to 9.5% (95% CI 8.4 to 10.7) of YA and 12.0% (95% CI 11.8 to 12.2) of adults. Cumulative incidence of LTFU after ART initiation at 5 years was 26.6% (24.6 to 28.6) among YA, 44.1% (41.8 to 46.3) among OA and 29.3% (29.1 to 29.6) among adults (Figure 1).

### 3.2 Correlates of LTFU after ART initiation

Thirty three of the 52 sites involved in this study had available site‐level data and their patients were included in these analyses, which represented 94.6% of patients within this dataset. In the multivariable model for the overall study population, the adjusted cause‐specific hazard ratio (aHR) of LTFU among OA was 1.54 (95% CI 1.41 to 1.68) (Table 3). By contrast, the hazard of LTFU was lower among YA compared to adults (aHR 0.77, 95% CI 0.69 to 0.86). When compared to men, the hazard of being LTFU was 10% lower among nonpregnant women (aHR 0.90, 95% CI 0.88 to 0.93) but 20% higher among pregnant women (aHR 1.20, 95% CI 1.14 to 1.27). In addition, more recent year of ART start was associated with higher hazard of LTFU. Patients with CD4 counts between 100 and 199 and 200 and 350 cells/μL had lower rates of LTFU compared to patients with CD4 count ≥350 cells/μL (aHR 0.86, 95% CI 0.81 to 0.90 and 0.82, 95% CI 0.77 to 0.86, respectively), while WHO stage 3 or 4 was associated with a 32% increased hazard of LTFU (aHR 1.32, 95% CI 1.28 to 1.36). Compared to participants who initiated ART in 2000 to 2004, aHR for LTFU among participants who initiated ART in 2010 to 2012 and 2013 to 2014 were 1.27 (95% CI 1.17 to 1.39) and 1.19 (95% CI 1.06 to 1.32), respectively.

**Table 3:** Cause‐specific hazard model of patient and site‐level correlates of LTFU after ART initiation among all patients, YA, OA and adults

Predictor	All patients	Young adolescents	Older adolescents	Adults
	aHR (95% CI)	aHR (95% CI)	aHR (95% CI)	aHR (95% CI)
Patient‐level characteristics				
Age group				
Young adolescents	0.77 (0.69 to 0.86)	‐	‐	‐
Older adolescents	1.54 (1.41 to 1.68)			
Adults	1.00			
Sex, pregnancy status				
Males	1.00	n/s	1.00	1.00
Nonpregnant females	0.90 (0.88 to 0.93)		1.51 (1.27 to 1.80)	0.89 (0.86 to 0.92)
Pregnant females	1.20 (1.14 to 1.27)		2.42 (1.98 to 2.95)	1.17 (1.10 to 1.24)
CD4 count at ART start (cells/μL)				
<100	1.02 (0.96 to 1.07)	n/s	n/s	1.02 (0.97 to 1.08)
100 to 199	0.86 (0.81 to 0.90)			0.86 (0.81 to 0.91)
200 to 349	0.82 (0.77 to 0.86)			0.81 (0.77 to 0.86)
≥350	1.00			1.00
WHO stage at ART start				
Stages I to II	1.00	1.00	n/s	1.00
Stages III to IV	1.32 (1.28 to 1.36)	1.34 (1.11 to 1.61)		1.33 (1.29 to 1.37)
Year of ART initiation				
2000 to 2004	1.00	1.00	n/s	1.00
2005 to 2009	0.97 (0.89 to 1.06)	0.81 (0.58 to 1.13)		0.98 (0.90 to 1.07)
2010 to 2012	1.27 (1.17 to 1.39)	1.21 (0.84 to 1.74)		1.29 (1.17 to 1.41)
2013 to 2014	1.19 (1.06 to 1.32)	3.43 (2.04 to 5.78)		1.21 (1.08 to 1.35)
Site‐level characteristics				
Programme				
AMPATH	1.00	1.00	1.00	1.00
FACES	0.93 (0.86 to 0.99)	0.46 (0.33 to 0.66)	0.48 (0.40 to 0.58)	0.94 (0.88 to 1.01)
MASAKA	0.96 (0.77 to 1.18)	0.43 (0.28 to 0.66)	0.59 (0.46 to 0.76)	0.92 (0.74 to 1.14)
MTCT‐PLUS	0.43 (0.35 to 0.52)	n/s	0.11 (0.02 to 0.78)	0.43 (0.35 to 0.53)
NACP	3.49 (3.02 to 4.03)	2.10 (0.61 to 2.76)	1.26 (0.96 to 1.66)	3.43 (2.96 to 3.98)
RAKAI	0.34 (0.25 to 0.45)	0.31 (0.04 to 2.18)	0.46 (0.25 to 0.83)	0.32 (0.24 to 0.43)
Level of care				
Primary	1.00	n/s	n/s	1.00
Secondary	1.09 (1.05 to 1.14)			1.09 (1.04 to 1.14)
Tertiary	0.61 (0.56 to 0.67)			0.62 (0.57 to 0.67)
Clinic setting and nutritional support				
Integrated clinics with food rations	0.93 (0.89 to 0.97)	n/s	n/s	0.93 (0.89 to 0.98)
Integrated clinics without food rations	1.02 (0.92 to 1.14)			1.04 (0.94 to 1.17)
Separate clinics with food rations	1.00			1.00
Support groups	0.77 (0.66 to 0.90)	n/s	n/s	0.77 (0.65 to 0.90)
Group adherence counselling	0.61 (0.57 to 0.67)	n/s	n/s	0.62 (0.57 to 0.67)
Peer educator programme	1.25 (1.18 to 1.31)	n/s	n/s	1.24 (1.18 to 1.31)
Outreach of patients missing visits	1.25 (1.20 to 1.39)	n/s	n/s	1.25 (1.12 to 1.39)

n/s, variable not statistically significant, excluded from multivariable model; aHR, adjusted hazard ratios; ART, antiretroviral therapy; CI, confidence interval; LTFU, loss to follow‐up

The hazard of LTFU in the overall study population varied widely among IeDEA programs. In comparison to the largest programme (AMPATH), all other programmes had lower hazard of LTFU, except the Tanzania NACP programme (aHR 3.49, 95% CI 3.02 to 4.03) (Table 3). Other site‐level factors associated with lower LTFU included: receiving care at tertiary versus primary‐care clinics (aHR 0.61, 95% CI 0.56 to 0.67), attending clinics with integrated adult and adolescent services and food rations (aHR 0.93, 95% CI 0.89 to 0.97), versus clinics without integrated services (but with food rations), and clinics having group adherence counselling (aHR 0.61, 95% CI 0.57 to 0.67), and patient support groups (aHR 0.77, 95% CI 0.66 to 0.90), versus not having these services. Interestingly, availability of peer educator programmes was associated with higher hazard of LTFU (aHR 1.25, 95% CI 1.18 to 1.31) as was having outreach programmes for missed visits, (aHR 1.25, 95% CI 1.20 to 1.39), compared to sites which did not have these programmes.

Results from age‐specific analyses for the YA, OA and adult populations are also shown in Table 3. Among OA, the hazard of LTFU was primarily driven by pregnant adolescents compared to adolescent males (aHR 2.42, 95% CI 1.98 to 2.95). Also among OA, in contrast to adult females, nonpregnant female adolescents had higher LTFU (aHR 1.51, 95% CI 1.27 to 1.80) compared to adolescent males. The variation with respect to programme of attendance among OA, YA and adults were consistent with those seen in the overall study population as described above. The correlates of LTFU among YA were similar to those observed in the overall study population, as described above.

## 4 Discussion

This analysis highlights the significantly higher LTFU after ART initiation among OA as compared to either YA or adults and is one of the first to identify the significant impact of pregnancy on LTFU among OA. Individual and structural reasons for such LTFU identified in the literature 13, 19, 20 could be amplified among OA due to limited autonomy and resources to independently continue ART. Our findings are in line with recent reports which suggest pregnancy at ART initiation is a significant risk factor for LTFU 8, 9, 10, 11, 12, and that postnatal disengagement from ART services is a significant programmatic challenge 8, 9, 21, 22. Our observations imply that pregnant OA initiating ART should be one of the key populations targeted for interventions to reduce LTFU in prevention of mother‐to‐child HIV transmission (PMTCT) programmes. Also notably, OA had the highest proportion of women pregnant at ART initiation, suggesting low rates of HIV testing or prior awareness of HIV positive status and/or recent HIV infections. This signals opportunities to intervene prior to early sexual encounters that result in pregnancy and HIV, and to engage adolescents in health care prior to pregnancy and HIV. Many pregnant OA may be starting ART soon after learning their HIV positive status, which in turn may impact ART retention as they deal with adjusting to the new diagnosis and lifelong treatment. In contrast to adults where nonpregnant female patients had lower hazard of LTFU compared to males, older adolescent girls who were not pregnant had over 50% higher hazard for LTFU compared to males. This suggests that, regardless of pregnancy status, adolescent girls should be included in key populations targeted for retention interventions.

We observed a worsening trend for LTFU among participants initiating ART in more recent years. This could mean inadequate ART retention in the face of significant scale‐up of ART services, and reflect challenges within health systems overwhelmed by an ever‐increasing ART population. It wasn't possible to account for undocumented transfers to other health facilities: this could have applied to some adolescents transitioning to adult care elsewhere 23, 24, 25. However, we expect that this would be a small proportion as all the health facilities in the analysis offered adolescent and adult care. Therefore, reasons for LTFU deserve additional qualitative exploration, particularly focusing ART access barriers and enablers for the pregnant OA population.

LTFU varied widely by programme in our analysis, which may reflect the impact of combination of programme‐specific characteristics, and the standard of care provided. Overall and by age‐group, LTFU was significantly lower at tertiary institutions. This may be a reflection of better resources typically provided at such institutions but is a concern in the wake of rapid ART‐scale up and decentralization to primary health facilities 26, 27. Our analysis also highlighted the importance of caring for adolescents and adults in integrated clinics. This is likely due to elimination of the “adult transition” step, which facilitates continued care and retention of adolescent records. While there is value in dedicated adolescent clinics 28, 29, 30, the transition period can prove a vulnerable point for LTFU 31. On the other hand, food support appeared to be a strong factor associated with reduction of LTFU among adults but not among adolescents (where integration of services was the main factor associated with reduced rates of LTFU). This is consistent with other studies on the subject and possibly delineates the different priorities and structural barriers to retention in these two age groups 30.

Although peer educator and outreach programmes are expected to be associated lower LTFU after ART initiation 32, 33, this association was has not observed among adults or adolescent ART programmes 34. Paradoxically, health facilities with these services had higher adult LTFU, and we cannot exclude the potential for reverse causality – perhaps sites which already had high LTFU preferentially implemented peer educator and outreach programmes to address patient retention issues in their programme 35.

The major strength of this study was the use of programmatic cohorts drawn from diverse real‐world clinical settings in East Africa, which making our findings generalizable to routine ART service delivery in our context. At the same time, our analysis has a number of limitations, including potential outcome misclassification as the result of passive collection of mortality data and incomplete documentation of “silent” transfers. Additionally, we determined LTFU at the time of database closure which could overestimate LTFU among participants newly initiating ART 36, as patients with longer ART observation times could have become disengaged and re‐engaged in care without being classified as LTFU 36. Finally, we had limited data on pregnant YA, and we only examined the impact of pregnancies at ART initiation (excluding pregnant women who initiated ART solely for PMTCT), and did not account for pregnancies occurring during ART follow‐up, all of which may have underestimated LTFU due to pregnancy. Despite these limitations, our analysis provides useful insights into the outcomes of the burgeoning adolescent population in our setting and describes a nuanced picture of LTFU correlates. These data can inform public health programmes to ensure the continuity and success of ART.

## 5 Conclusion

OA initiating ART experienced the highest rates of LTFU when compared to YA and adults. Among the OA, females (pregnant or not) had particularly high risk of being LTFU. This analysis highlights the need to develop, test and assess interventions designed to retain older adolescents on ART, particularly girls and women initiating ART while pregnant.

## Competing interests

None. The authors report no conflicts of interest.

## Authors’ contributions

HNB, BE, ANK, CTY, KWK and EJA contributed to the conception of the analysis idea; BSM, ANK, HNB, CTY, SA, HF, EL and JS contributed to data collection and cleaning; ANK, BSM and CTY analysed the data; HNB, BE, ANK and CTY wrote the manuscript; HNB, BE, ANK, CTY, KWK, BSM, EJA, SA, HW, EL and JS reviewed and contributed to the manuscript; BE gave overall technical oversight for the analytic process and manuscript writing.
